# Serum-biomarker-based population screening model for hepatocellular carcinoma

**DOI:** 10.1016/j.isci.2025.111981

**Published:** 2025-02-08

**Authors:** Wenmin Liao, Wenbin Lin, Zhonglian He, Chenyang Feng, Yuying Liu, Zixian Wang, Ruizhi Wang, Meifang He, Shuqin Dai, Ying Sun, Wei Wei, Peisong Chen, Chaofeng Li

**Affiliations:** 1 State Key Laboratory of Oncology in South China, Guangdong Provincial Clinical Research Center for Cancer, Sun Yat-sen University Cancer Center, Guangzhou 510060, P.R. China; 2 Department of Information Technology, Sun Yat-sen University Cancer Center, Guangzhou 510060, P. R. China; 3 Department of Clinical Laboratory, The First Affiliated Hospital of Sun Yat-Sen University, Guangzhou 510080, China; 4 Department of Medical Oncology, Sun Yat-sen University Cancer Center, Guangzhou 510060, P. R. China; 5 Laboratory of General Surgery, The First Affiliated Hospital, Sun Yat-Sen University, Guangzhou 510080, P. R. China; 6 Department of Clinical Laboratory, Sun Yat-Sen University Cancer Center, Guangzhou 510060, China; 7 Department of Radiation Oncology, Sun Yat-Sen University Cancer Center, Guangzhou 510060, P. R. China; 8 Department of Hepatobiliary Oncology, Sun Yat-sen University Cancer Center, Guangzhou 510060, China

**Keywords:** Public health, Cancer

## Abstract

Hepatocellular carcinoma (HCC) early identification is crucial for improving patient outcomes. Current screening methods are often complex and costly. This study developed a simplified, cost-effective HCC screening model using serum marker data. A diverse study population from two Chinese hospitals was recruited, including cancer patients, hospital patients, and healthy individuals. A two-stage screening model was created: LASSO logistic regression for preliminary screening, followed by logistic regression incorporating alpha-fetoprotein (AFP). The model’s performance was evaluated in multiple cohorts. Across five populations, the model showed strong performance with AUC-ROC ranging from 0.868 to 0.907, accuracy between 87.43% and 96.96%, and sensitivity over 75% with specificity above 90%. Compared with solely AFP models, the second-stage model improved HCC risk estimates in healthy populations, with significantly higher AUC (0.930 vs. 0.827) and net reclassification improvement (NRI) up to 56.2%. This two-stage model offers a practical, cost-efficient tool for early HCC detection, addressing a significant public health need.

## Highlights

• The study developed a two-stage HCC screening model using serum biomarker
• The model shows high accuracy in identifying HCC risk across multiple cohorts
• The model provides a cost-efficient tool for early HCC detection

## Graphical abstract

**Figure:**
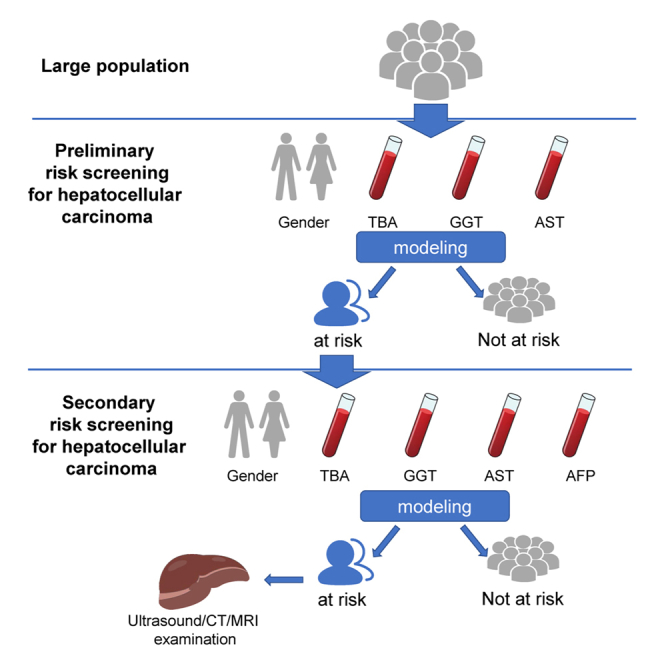

## Introduction

Hepatocellular carcinoma (HCC), the most prevalent primary liver malignancy, accounts for approximately 85%–90% of all primary liver cancers and stands as the third leading cause of cancer-related death worldwide, claiming an estimated 745,500 patients annually.^1^^,^^2^ Despite considerable advancements in diagnostic and therapeutic strategies over the past three decades, the prognosis of HCC remains largely unimproved.^3^^,^^4^ A significant contributing factor is the lack of reliable biomarkers for early detection, compounded by considerable economic challenges in effective diagnosis and treatment among high-risk populations,^5^ which resulted in the majority of HCC cases that are still diagnosed based on symptomatic presentation, rather than proactive early screening and detection.

Health screening refers to identify a subset of asymptomatic individuals in the early stages of disease or those at elevated risk, thereby enabling early diagnosis and treatment to improve disease prognosis.^6^ Ideal techniques for HCC population screening should exhibit cost-effectiveness, ease of operation, and widespread applicability (without causing physical harm), while maintaining adequate diagnostic accuracy. Additionally, when designing screening protocols, it is crucial to consider the potential psychological impact of false-positive results, which can lead to significant emotional distress for affected individuals. Therefore, the development of HCC screening strategies must prioritize both high sensitivity and high specificity to minimize both missed and erroneous diagnoses.

Currently, available methods for HCC diagnosis include serological examinations, imaging modalities, and histological biopsies.^5^^,^^7^^,^^8^ Various imaging techniques, such as computed tomography (CT), magnetic resonance imaging (MRI), and ultrasound, have significantly improved the sensitivity of HCC diagnosis from 66% to 82%. Additionally, the specificity for detecting nodules larger than 1 cm in diameter has increased to over 90%.^9^ In line with this, the National Comprehensive Cancer Network (NCCN) clinical practice guidelines recommend semi-annual abdominal ultrasound and alpha-fetoprotein (AFP) screening for individuals at high risk of HCC, especially those with cirrhosis of any etiology, to closely monitor disease progression.^10^^,^^11^ However, it is important to note that the diagnostic accuracy of imaging heavily relies on physician expertise, often necessitating the integration of artificial intelligence interventions such as radiomics to achieve a more precise diagnosis.^7^ Moreover, although MRI offers high sensitivity, its routine use for surveillance is economically prohibitive, limiting its feasibility for large-scale HCC monitoring.^12^

In recent years, there has been a paradigm shift in HCC population screening from a one-size-fits-all approach to individualized HCC risk assessment, driven by advancements in serological biomarkers.^13^ Therefore, combining multiple serum biomarkers to form a comprehensive score as a screening strategy holds significant value in reducing missed and misdiagnosis. A previous cross-country cohort study comprising 2,400 individuals found that the combined “GALAD” score, encompassing gender, age, AFP, AFP-L3, and PIVKA-II, exhibited relatively high sensitivity for both random and early-stage HCC screening, achieving over 70% and 60% sensitivity, respectively.^14^ Furthermore, a multicenter retrospective study in China developed the “ASAP” scores, incorporating age, gender, AFP, and PIVKA-II among hepatitis-B-virus-infected individuals. When compared to the GALAD score through a multicenter case-control study, the ASAP score demonstrated superior diagnostic performance for the early detection of HCV-HCC.^15^ However, it is important to note that neither the GALAD nor ASAP scores have been validated for screening in large natural populations, and current standard screening strategies for mass population health screening remain inconclusive. Additionally, the limited availability of PIVKA-II and AFP-L3 testing in developing countries like China poses significant challenges to the widespread implementation of these models in large-scale population screening.

Therefore, there remains a significant gap in HCC screening methods. Given the ongoing trend, it is evident that until more specific biological markers for HCC are discovered, screening models incorporate commonly available blood biomarkers using extensive population samples and offer a balance between diagnostic accuracy and economic feasibility. This makes them highly suitable for implementation and dissemination within HCC prevention and control strategies. In light of this context, the current study is devised to create a risk prediction model for the diagnosis and monitoring of HCC, utilizing extensive population data. Additionally, the study strives to thoroughly assess the model’s stability and practicality in real-world scenarios, with the objective of promoting its utilization in the early identification of HCC.

## Results

**Figure 1:**
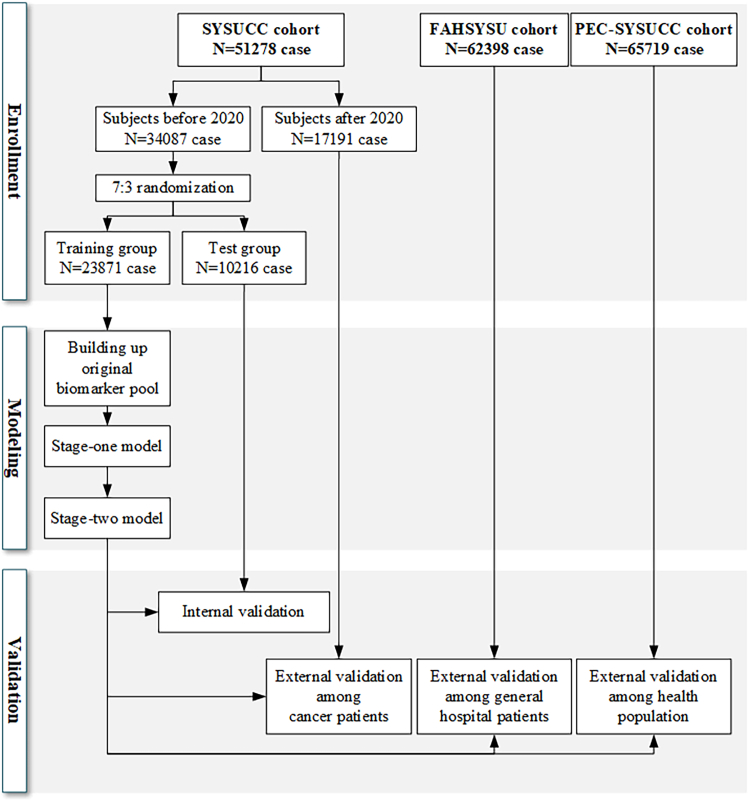
Study cohorts and statistical analyses workflow

The present study comprised a total of 179,395 individuals, including 38,027 HCC cases and 141,348 controls, sourced from five distinct cohorts: training group (23,871 individuals), test group (10,216 individuals), cancer patients (17,191 individuals), general patients (62,398 individuals), and healthy population (65,719 individuals) (Figure 1). Table 1 outlines the demographic and clinical characteristics of the enrolled subjects across these cohorts, specifically focusing on age, gender, total bile acid (TBA), aspartate aminotransferase (AST), gamma-glutamyl transferase (GGT), and alpha-fetoprotein (AFP). The median age of the overall study population was 53 years, with a notable trend indicating that patients in the SYSUCC cohort were older compared to those in the FAHSYSU and PEC-SYSUCC cohorts. A significant gender disparity was observed, with a higher proportion of males among HCC patients consistent across all cohorts. Specifically, the male proportion among HCC patients was 87%, 89%, and 88% in the SYSUCC, FAHSYSU, and PEC-SYSUCC cohorts, respectively, compared to 49%, 54%, and 52% in the corresponding healthy control groups. Furthermore, the TBA, GGT, AST, and AFP levels in HCC patients were consistently elevated compared to those of healthy individuals, both in the overall population and within each cohort.

**Table 1:** Population characteristic of enrolled subjects

Characteristic	Overall			SYSUCC cohort						FAHSYSU cohort		PEC-SYSUCC cohort	
				Training group		Test group		Cancer patients		General patients		Healthy population	
	Overall, *N* = 179,395	HCC patients, *N* = 38,047	Controls, *N* = 141,348	HCC patients, *N* = 6,452	Controls, *N* = 17,419	HCC patients, *N* = 2,785	Controls, *N* = 7,431	HCC patients, *N* = 2,533	Controls, *N* = 14,658	HCC patients, *N* = 26,241	Controls, *N* = 36,157	HCC patients, *N* = 36	Controls, *N* = 65,683
Age	53 (43, 64)	56 (47, 65)	52 (42, 63)	61 (52, 69)	66 (55, 73)	61 (52, 69)	66 (56, 73)	57 (49, 65)	59 (51, 68)	54 (46, 63)	48 (40, 55)	54 (43, 66)	48 (38, 59)
Gender													
Male	107,231 (60%)	33,715 (89%)	73,516 (52%)	5,606 (87%)	8,815 (51%)	2,394 (86%)	3,818 (51%)	2,217 (88%)	6,913 (47%)	23,464 (89%)	19,622 (54%)	34 (94%)	34,348 (52%)
Female	72,164 (40%)	4,332 (11%)	67,832 (48%)	846 (13%)	8,604 (49%)	391 (14%)	3,613 (49%)	316 (12%)	7,745 (53%)	2,777 (11%)	16,535 (46%)	2 (5.6%)	31,335 (48%)
TBA	3 (2, 6)	9 (5, 20)	3 (2, 5)	7 (4, 14)	3 (2, 5)	7 (4, 15)	3 (2, 5)	7 (4, 16)	3 (2, 6)	10 (5, 23)	2 (2, 4)	5(3, 9)	3 (2, 5)
GGT	26 (17, 51)	83 (42, 170)	22 (15, 35)	70 (39, 140)	23 (16, 35)	71 (39, 139)	22 (16, 35)	75 (39, 160)	22 (15, 34)	89 (44, 183)	23 (16, 36)	46 (25, 93)	22 (15, 34)
AST	21 (17, 29)	41 (28, 69)	20 (16, 24)	37 (27, 57)	19 (16, 24)	38 (27, 58)	19 (16, 24)	37 (27, 60)	18 (15, 23)	43 (29, 75)	22 (19, 26)	28 (19, 51)	19 (16, 23)
AFP	4 (2, 13)	69 (6, 1,563)	3 (2, 4)	63 (6, 1,129)	3 (2, 4)	55 (6, 1,009)	3 (2, 4)	49 (5, 790)	3 (2, 4)	76 (6, 1,941)	3 (2, 4)	5 (4, 9)	3 (2, 4)
Unknown	89,139	4,272	84,867	350	10,997	166	4,653	153	5,916	3,603	6,741	0	56,560

Age, TBA, GGT, AST, and AFP are expressed as median (interquartile range); other data are expressed as number (percentage). The number of missing AFP is listed as “unknown” due to its low detection rate.

TP, total bile acid; GGT, gamma-glutamyl transpeptidase; AST, aspartate transferase; AFP, alpha-fetoprotein; HCC, hepatocellular carcinoma.

### Development of a preliminary risk screening model for HCC

Following the calculation of population-based deletion rates for each biomarker in the modeling cohort, a pool of 22 potential risk factors was established as the original biomarker pool (OBP). These factors were subsequently integrated into an LASSO logistic regression model. Table S1 details the allocation of variables derived from the OBP. To optimize the LASSO logistic model’s performance, we employed generalized cross-validation. The area under the curve (AUC) of the model varied with changes in the log (λ) value of the tuning parameter. The number of variables selected by the LASSO logistic model is graphically represented in Figure S1. By setting log (λ) = −2.8, we minimized the number of variables while maximizing predictive accuracy.

After the LASSO logistic analysis, four risk factors emerged as significant: gender, AST, GGT, and TBA. The ranking of the indicators by importance is as follows: AST, GGT, TBA, and gender, with absolute coefficients being 7.52, 2.86, 0.78, and 0.42, respectively. These factors were incorporated into the preliminary risk screening model (stage-one model), formulated as:

$${{Prob}_{1}}_{\left(HCC=1\right)}=-6.301-1.428\times \text{Gender}+3.002\times \text{TBA}+4.001\times \text{GGT}+12.307\times \text{AST}$$

**Figure 2:**
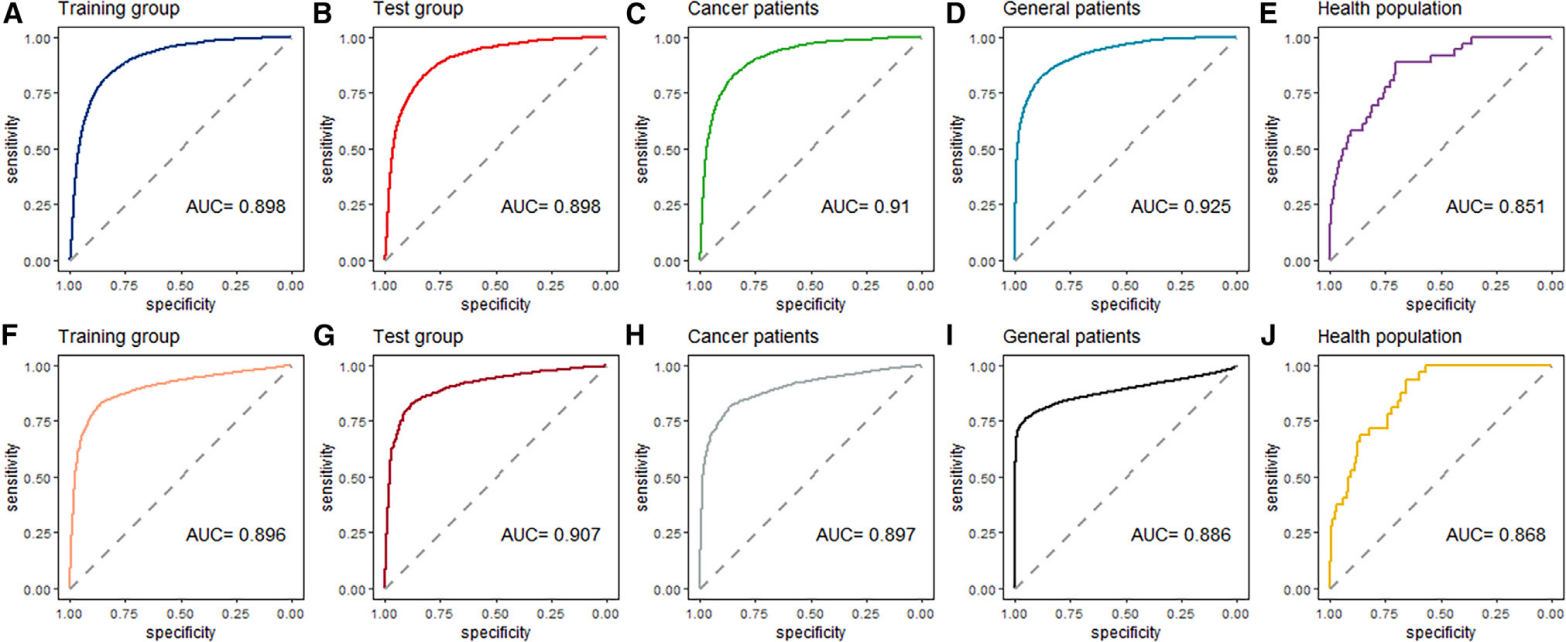
ROC curves of the two-stage model in each cohort (A–E) The preliminary risk screening model; (F–J) the secondary risk screening model. Data are represented as mean ± SEM.

The ROC curves of the stage-one model in all study cohorts were shown in Figures 2A–2E; the AUC values for the training group, the test group, the cancer patients, the general patients, and the healthy population were 0.898 (95% confidential interval [CI]: 0.894–0.903), 0.898 (95% CI: 0.891–0.905), 0.910 (95% CI: 0.904–0.916), 0.925 (95% CI: 0.923–0.927), and 0.851 (95% CI: 0.791–0.910), respectively.

Using a grid-search algorithm in the training group, we identified a cutoff value of 0.184, which maximized sensitivity (>90%) while optimizing the sum of sensitivity and specificity. This cutoff, designated as ${\text{cutoff}}_{1}$, was used to classify individuals as high-risk based on their ${{Prob}_{1}}_{\left(HCC=1\right)}$ scores from the stage-one model. Individuals with scores exceeding 0.184 were considered preliminary high-risk and progressed to the stage-two model for further screening.

### Development of a secondary risk screening model for HCC

Among the preliminary screened high-risk population, an additional biomarker, AFP, was incorporated alongside the four variables identified in the first stage. Utilizing logistic regression analysis, a secondary screening model was formulated based on these five independent variables. The structure of this model, tailored for the initially screened risk population, is as follows:

$${{Prob}_{2}}_{\left(HCC=1\right)}=-3.626-1.372\times \text{Gender}+1.258\times \text{TBA}+1.065\times \text{GGT}+6.319\times \text{AST}+12.129\times \text{AFP}$$

The diagnostic performance of the second-stage model was evaluated across various study cohorts, and the corresponding ROC curves are presented in Figures 2F–2J. The AUC values obtained for the training group, test group, cancer patients, general patients, and healthy population were 0.896 (95% CI: 0.888–0.904), 0.907 (95% CI: 0.895–0.918), 0.897 (95% CI: 0.887–0.907), 0.886 (95% CI: 0.882–0.890), and 0.868 (95% CI: 0.820–0.916), respectively.

The optimal cutoff value for the second-stage model, designated as ${\text{cutoff}}_{2}$, was determined to be 0.417. Individuals with a ${{Prob}_{2}}_{\left(HCC=1\right)}$ score exceeding 0.417 were labeled to be the final individuals at risk of HCC screened by the two-stage model.

### Screening efficiency of the two-stage model for HCC

The screening efficiencies of the two-stage screening model across various subpopulations are meticulously presented in Table 2. Notably, the accuracy of this model ranges between 87.43% and 97.00% across all study cohorts, highlighting its reliability and consistency. Moreover, all reported values of sensitivity exceed 75%, whereas specificity values surpass 90%.

**Table 2:** Performance of the two-stage screening model in diagnosing HCC

Study cohort		Sensitivity (%)	Specificity (%)	NPV (%)	PPV (%)	Accuracy (%)
Modeling cohort	Training group	84.64	93.26	93.43	84.29	90.68
	Test group	85.16	93.04	93.60	83.99	90.68
Cohort of cancer patients		82.96	91.63	96.77	64.06	90.31.
Cohort of general patients		77.28	94.13	86.25	89.70	87.43
Cohort of healthy population		88.89	97.01	99.99	2.16	97.00

NPV, negative predictive value; PPV, positive predictive value.

**Figure 3:**
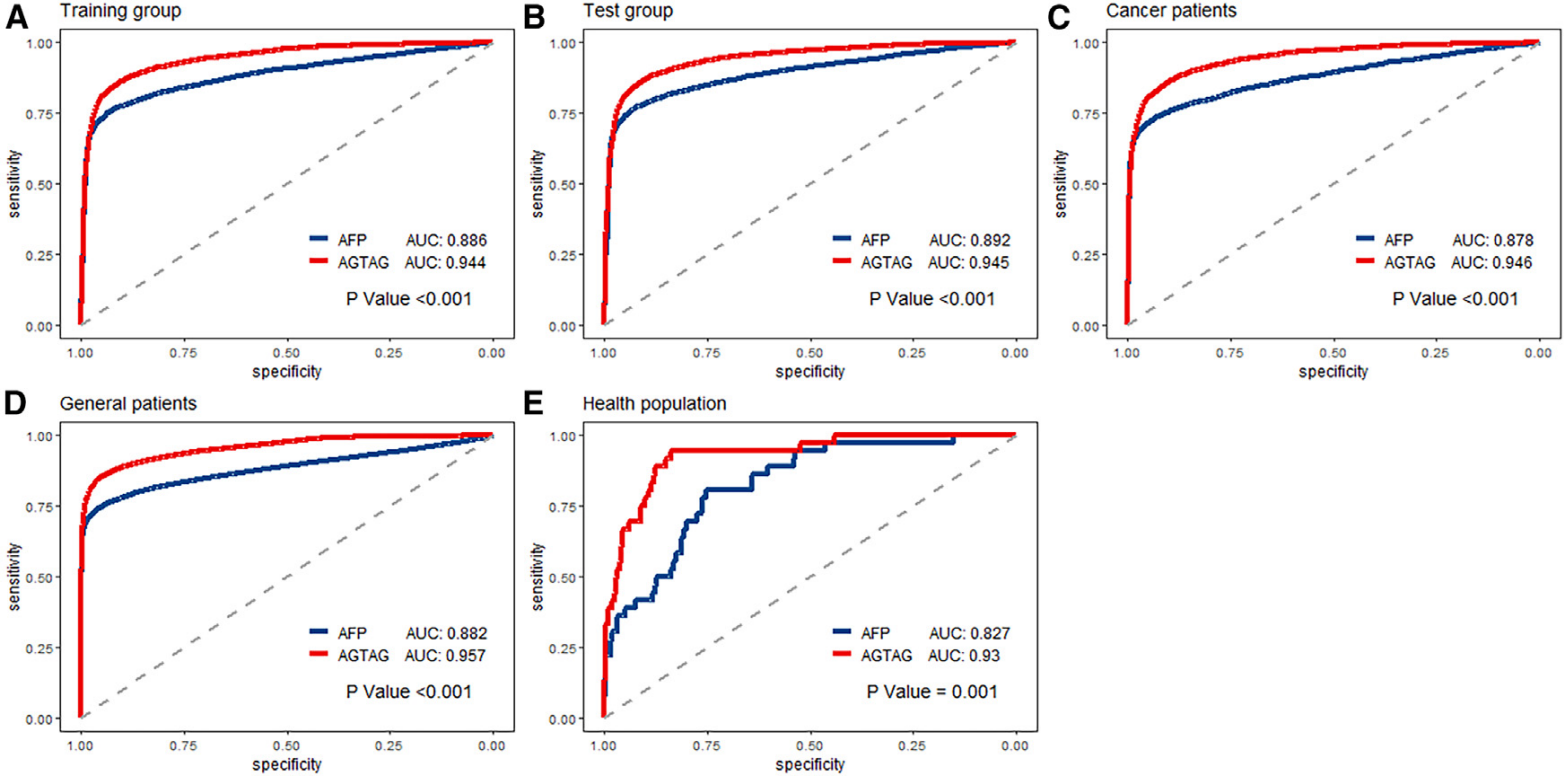
Screening performance of utilizing solely AFP versus AGTAG in each cohort (A–E) ROC curves of solely AFP and AGTAG in each cohort. Data are represented as mean ± SEM. *p* values are evaluated using the DeLong tests.

To further elucidate the efficacy of this two-stage model, Figure 3 showed the screening performance of utilizing solely AFP as a screening variable versus employing the second-stage model (designated as “AGTAG”). As shown in Figure S2, the decision curve analysis indicated that when the threshold probabilities ranged between about 0.5% and 80% in each cohort, the use of the “AGTAG” model to HCC risk provided greater net benefit than the “treat all,” “treat none,” or “AFP” strategies, which indicates the clinical usefulness of the “AGTAG” model. Intriguingly, the “AGTAG” approach exhibits significantly superior performance compared to AFP alone in all study cohorts, including the healthy population (*p* < 0.001). Among healthy population, compared with the model using only AFP and setting the clinical reference (25 ng/mL) as the cutoff value or setting the optimal cutoff value based on the Youden index criterion, the AGTAG model had improved accuracy in risk estimates. The net reclassification improvement (NRI) (95% CI) values were 56.2% (42.0%, 70.3%) and 19.80% (6.1%, 33.5%) (Table 3).

**Table 3:** Net reclassification improvement of screening model in healthy population

Truly outcome: non-HCC				Truly outcome: HCC			Combined data			Overall net reclassification improvement (95% CI)	*P* for net reclassification improvement
AGTAG model			Reclassified percentage	AGTAG model		Reclassified percentage	AGTAG model		Reclassified percentage		
	0	1		0	1		0	1			
**AFP model**^a^											

0	7390	1719	19%	2	27	93%	7392	1746	19%	56.2% (42.0%, 70.3%)	<0.001
1	0	14	0%	0	7	0%	0	21	0%		

**AFP model**^b^											

0	6146	704	10%	1	6	86%	6147	710	10%	19.8% (6.1%, 33.5%)	0.005
1	1244	1029	55%	1	28	3%	1245	1057	54%		

a Setting clinical reference (20 ng/mL) as cutoff value.

b Setting the optimal cutoff value based on the Youden index criterion.

For the convenience of clinicians and the public in self-assessment, we have developed an online calculator for hepatocellular carcinoma risk, which can be accessed via the website at http://hccscreening.sysucc.org.cn:2401/AGTAG_en.html.

## Discussion

In the present investigation, a two-stage screening model for HCC was devised, and its efficacy was assessed in multiple cohorts, collectively comprising over 170,000 individuals. This study represents, to the authors' knowledge, the most extensive real-world study of liver cancer screening conducted thus far. The screening approach is characterized by its dual-phase structure. The initial screening utilizes solely gender and three serum markers (AST, GGT, TBA) for preliminary assessment, whereas the subsequent phase incorporates an additional serum marker, AFP, for further evaluation. This strategic design ensures both cost-effectiveness and feasibility in large-scale population screening scenarios.

The five risk factors identified in the two-stage screening model (gender, TBA, GGT, AST, and AFP) have all been previously demonstrated associated with HCC. Gender differences in HCC incidence have been well documented,^13^ with men exhibiting higher prevalence rates. Recent research has implicated hormonal factors, such as testosterone-mediated suppression of adiponectin, in the pathogenesis of HCC.^16^ AFP, a long-standing biomarker in HCC screening, has been included in various diagnostic guidelines.^17^^,^^18^ Despite its limitations, AFP remains a widely used tool in HCC surveillance due to its association with disease progression.^19^ The ranking of the indicators suggests that changes in AST levels have the greatest impact on the log-odds of the outcome variable. Other serum markers, including GGT and TBA, have also been linked to liver dysfunction and HCC development.^20^ These markers, which are routinely measured in clinical practice, provide valuable insights into liver function and potential abnormalities.

By incorporating these markers into the screening model, the study has demonstrated the feasibility of using routinely available clinical data for the early identification of individuals at risk of HCC. Notably, the two-stage model exhibited consistent and remarkable performance across diverse cohorts, including cancer patients, general patients, and healthy individuals. This consistency suggests its potential utility in population-wide HCC screening.

Currently, HCC diagnosis often relies on a combination of serological tests, imaging modalities, and histological biopsies.^5^^,^^7^^,^^8^ It is important to acknowledge the role of AFP, AFP-L3, and PIVKA-II in HCC screening. However, serological tests that employ single serum markers often suffer from limited sensitivity and specificity.^21^ In particular, AFP, a widely used biomarker in liver cancer diagnosis, has a sensitivity of only 62.4% at the diagnostic threshold of 20 ng/mL, rendering it insufficient for early detection and prone to false negatives.^5^^,^^22^ The current study also demonstrated that AGTAG model has better efficacy than AFP in the screening of HCC in healthy people, which is strong evidence of the efficacy of this HCC screening method in the real world and can provide a valuable reference for future pan-cancer blood screening programs.

Although AFP-L3 and PIVKA-II have been recognized as classical indicators for HCC alongside AFP,^14^ during our data collection process, we observed that PIVKA-II and AFP-L3 are not consistently available across all medical institutions and are frequently omitted from routine health check-ups because of higher economic cost.^23^ As a result, constraints in data availability prevented us from incorporating these biomarkers into our model. However, this exclusion may not necessarily be a drawback. Our study aimed to develop a streamlined and easily accessible model that could be widely applied to large populations. By utilizing commonly used liver function indicators (preliminary risk screening model) and additional AFP test (secondary risk screening model), we believe we can still achieve a good screening effect. This approach enhances the economic and operational feasibility of large-scale HCC screening, making it more practical and accessible for widespread implementation.

In recent years, there has been a growing trend toward utilizing combinations of serum markers for HCC detection. Models such as GALAD, BALAD, and ASAP have been developed to calculate individualized HCC risk.^24^^,^^25^^,^^26^ Although these models have shown promise in risk stratification for patients with chronic liver diseases,^25^^,^^26^^,^^27^^,^^28^^,^^29^^,^^30^^,^^31^ they differ from the present two-stage model, which is tailored for mass population screening. The current model’s reliance on easily accessible serum markers and its simplicity make it particularly suitable for resource-limited settings with a high HCC burden.^31^

In summary, the two-stage screening model introduced in this study provides a cost-effective and practical solution for large-scale HCC screening. Its straightforward design and dependence on readily available serum markers enhance its attractiveness for deployment in resource-constrained environments. The consistent effectiveness of this model across diverse cohorts further highlights its potential utility in reducing the global burden of HCC.

### Limitations of the study

The study has several limitations. Firstly, the retrospective nature of the study may have caused the results to be influenced by potential biases that were not measured. However, the results we obtained in a total population of about 170,000 people can be used as a real-world evidence and provide answers relevant to broader population.^32^ In the future, it may be possible to validate the performance of this screening model with more prospective studies. Secondly, the screening efficiency of the model is greatly affected by different prevalence rates. Due to the low prevalence of HCC among healthy population, the positive predictive value in the healthy population cohort was low. Although the high accuracy of this model in healthy people reduces the harm of misdiagnosis to a certain extent, future studies can also explore population subgroups in which this model can achieve higher efficacy and further improve the accuracy of screening. Thirdly, our two-stage model did not include individuals with underlying liver diseases, such as cirrhosis and hepatitis, as a control group. This limits the generalizability of our findings to these high-risk populations, who may require more specialized screening approaches. Future research should focus on developing and validating models specifically tailored for individuals with pre-existing liver conditions to enhance the precision of HCC screening in these populations.

## Resource availability

### Lead contact

Further information and requests for resources and data should be directed to and will be fulfilled by the lead contact, Dr. Chaofeng Li (lichaofeng@sysucc.org.cn).

### Materials availability

This study did not generate new unique reagents.

### Data and code availability

• The datasets used and/or analyzed during the current study are available from the corresponding author on reasonable request.
• This paper does not report original code.
• Any additional information required to reanalyze the data reported in this paper is available from the lead contact upon request.

## Acknowledgments

This work was supported by 10.13039/501100001809National Natural Science Foundation of China (8217101860).

## Author contributions

C.L. and W.Liao. designed the study and drafted the paper; C.L., W.Liao., W.Lin., Z.H., P.C., and W.W. prepared and analyzed the data. C.F., Z.W., R.W., M.H., S.D., and Y.S. assisted with interpretation of the data, edited on drafts of the manuscript.

## Declaration of interests

The authors declare that they have no competing interests.

## STAR★Methods

### Key resources table

**Table:** 

REAGENT or RESOURCE	SOURCE	IDENTIFIER
**Biological samples**		

Serum Marker	the laboratory information system of respective hospitals	SYSUCC: COBAS 800 e702 module (Roche Diagnostics) for biochemical indexes; COBAS e 801 (Roche Diagnostics) for AFP. FAHSYSU: AU5800 Series Chemistry Analyzers (Beckman Coulter) for biochemical indexes; Alinity immunoassay analyser (Abbott Diagnostics) for AFP.

**Software and algorithms**		

R version 3.6.1	https://www.r-project.org/	R packages ‘glmnet’, ‘pacman’, ‘pROC’, ‘PredictABEL’, ‘rmda’, and ‘ggplot2’.

### Experimental model and study participant details

#### Ethics approval

The multicenter retrospective study was granted ethical approval by both the ethics committee of Sun Yat-Sen University Cancer Center (No. B2023-486-01) and the ethics committee of the First Affiliated Hospital of Sun Yat-Sen University (No. 2020-339). The requirement for informed consent was waived by both ethics committees due to the retrospective nature of the study. Stringent measures were taken to anonymize all personal data and ensure its analysis on a population scale, thereby protecting patient privacy.

#### Study population

The study population comprised two groups: HCC patients, diagnosed with hepatocellular carcinoma, and controls, lacking any clinical, histological, or radiological diagnosis of liver disease. Subjects were identified retrospectively from three institutions in Guangzhou, China: Sun Yat-Sen University Cancer Center (SYSUCC), Physical Examination Center of SYSUCC (PEC-SYSUCC), and the First Affiliated Hospital of Sun Yat-Sen University (FAHSYSU).

#### Study period

The study period spanned from January 1st, 2010, to December 31st, 2022. For subjects from SYSUCC and FAHSYSU, diagnostic information was retrieved from the electronic medical record system and individuals were labeled as ‘HCC case’ or ‘control’. For subjects from PEC-SYSUCC, diagnostic information was obtained through follow-up survey.

### Method details

#### Serum marker measurement

All serum marker measurements were obtained from the laboratory information system of the respective hospitals. At SYSUCC, biochemical indexes were measured using the COBAS 800 e702 module (Roche Diagnostics, Tokyo, Japan) and the AFP was measured using the COBAS e 801 analytical unit (Roche Diagnostics, Tokyo, Japan). At FAHSYSU, biochemical indexes were measured using the AU5800 Series Chemistry Analyzers (Beckman Coulter, USA) and the AFP was measured using the Alinity immunoassay analyser (Abbott Diagnostics, Chicago, USA). Initially, the study applied a logarithmic transformation to positively-skewed measurements and retains the original values of normally-distributed measurements (Table S1). Subsequently, Min-Max normalization^33^ is employed to mitigate measurement disparities across institutions, ensuring data comparability. Prediagnostic measurements were defined as the values closest to the time of HCC diagnosis within four weeks before diagnosis. For HCC patients, prediagnostic measurements were collected, while for controls, their first measurements were recorded.

#### Study cohorts

The subjects were divided into several cohorts for analysis. HCC patients and control from SYSUCC, enrolled up to December 31st, 2019, were combined to form a modeling cohort. This cohort was randomly divided into a training group and a test group with a 7:3 ratio. Internal validation was conducted in the test group after modeling the training group. Additionally, three external validation cohorts were established: patients from SYSUCC enrolled between January 1st, 2020, and December 31st, 2022; patients from FAHSYSU enrolled since January 1st, 2010; and guests from PEC-SYSUCC enrolled between June 19th, 2012, and December 31st, 2022. These cohorts aimed to evaluate the model's performance across cancer patients, general hospital patients, and the healthy population (Figure 1).

### Quantification and statistical analysis

#### Original biomarker pool

Considering both cost-effectiveness and universality, the demographic characteristics and serum markers selected for population screening should be easily accessible. We calculated the population deletion rate for each demographic characteristic and serum marker in the modeling cohort to ensure their inclusion was feasible. Those demographic characteristics and serum markers with a population deletion rate of less than 10% were included in an original biomarker pool (OBP).

#### Model development

A two-stage screening model was developed for the training group. In the first stage, the least absolute shrinkage and selection operator (LASSO) regression was applied to select variables relevant to HCC diagnosis.^34^^,^^35^ HCC diagnosis was used as the dependent variable, and biomarkers from the OBP were preliminary screening through LASSO logistic regression analysis as risk factors contributing to HCC diagnosis. After deriving the simplest logistic regression model (referred to as the preliminary risk screening model), to ensure high sensitivity in population screening, a grid-search algorithm was employed to determine a threshold value with sensitivity exceeding 90% and the highest sum of sensitivity and specificity as the final cutoff value for the risk screening model (designated as ${\text{cutoff}}_{1}$). Subjects with a predicted probability exceeding ${\text{cutoff}}_{1}$ were labeled as ‘at risk’ in the first stage.

Recognizing that the first-stage risk screening model sacrificed specificity for high sensitivity, resulting in many healthy individuals being incorrectly classified as ‘at risk’, a secondary screening was conducted within the preliminary screened risk group. In the second stage of the model, an additional variable, AFP, was incorporated into the logistic model to further assess patient risk. ROC curves were plotted, and an optimal cutoff value (${\text{cutoff}}_{2}$) was determined based on the Youden index criterion.^36^ This approach ensured optimal diagnostic accuracy of the two-stage model, thereby enhancing its reliability and practicality in clinical settings. Subjects with a risk degree exceeding ${\text{cutoff}}_{2}$ were identified as the final HCC risk individuals.

#### Model evaluation

After establishing the two-stage model, its efficacy was evaluated across multiple cohorts, comprising testing group, cancer patients, general hospital patients, as well as healthy population. The sensitivity and specificity of the two-stage population screening model in each study cohort were calculated and ROC curve for each stage in each cohort was plotted.

Furthermore, ROC curves and decision curve analysis (DCA)^37^ were constructed for both the logistics regression model using AFP alone and the second-stage model in different cohorts, comparing their screening effectiveness and validity. Subsequently, the observed differences between these ROC curves were statistically evaluated using the DeLong test.^38^ In the screening of healthy population of interest, the net reclassification improvement (NRI)^39^ was estimated to quantify the effectiveness of the second-stage model in reclassifying individuals in terms of predicted liver cancer risk compared to the AFP model alone, which was based on clinical reference values as cut-off and the optimal cut-off set according to the Youden index.

#### Software and significance level

All analyses were conducted using R version 3.6.1, packages including with ‘glmnet’, ‘pacman’, ’pROC’, ‘PredictABEL’, ‘rmda’ and ‘ggplot2’. Results were considered statistically significant at *P*<0.05.

### Additional resources

For the convenience of clinicians and the public in self-assessment, we have developed an online calculator for hepatocellular carcinoma risk, which can be accessed via the website at: http://hccscreening.sysucc.org.cn:2401/AGTAG_en.html.
