# Supplementary material for: Serum-biomarker-based population screening model for hepatocellular carcinoma
Source: iScience. 2025 Feb 8;28(3):111981. doi: 10.1016/j.isci.2025.111981 (PMC11889663; doi:10.1016/j.isci.2025.111981)
Supplement: Document S1. Figures S1 and S2 and Table S1 [file mmc1.pdf]

iScience, Volume *28*

Supplemental information

Serum-biomarker-based population screening model for hepatocellular carcinoma

Wenmin Liao, Wenbin Lin, Zhonglian He, Chenyang Feng, Yuying Liu, Zixian Wang, Ruizhi Wang, Meifang He, Shuqin Dai, Ying Sun, Wei Wei, Peisong Chen, and Chaofeng Li

G

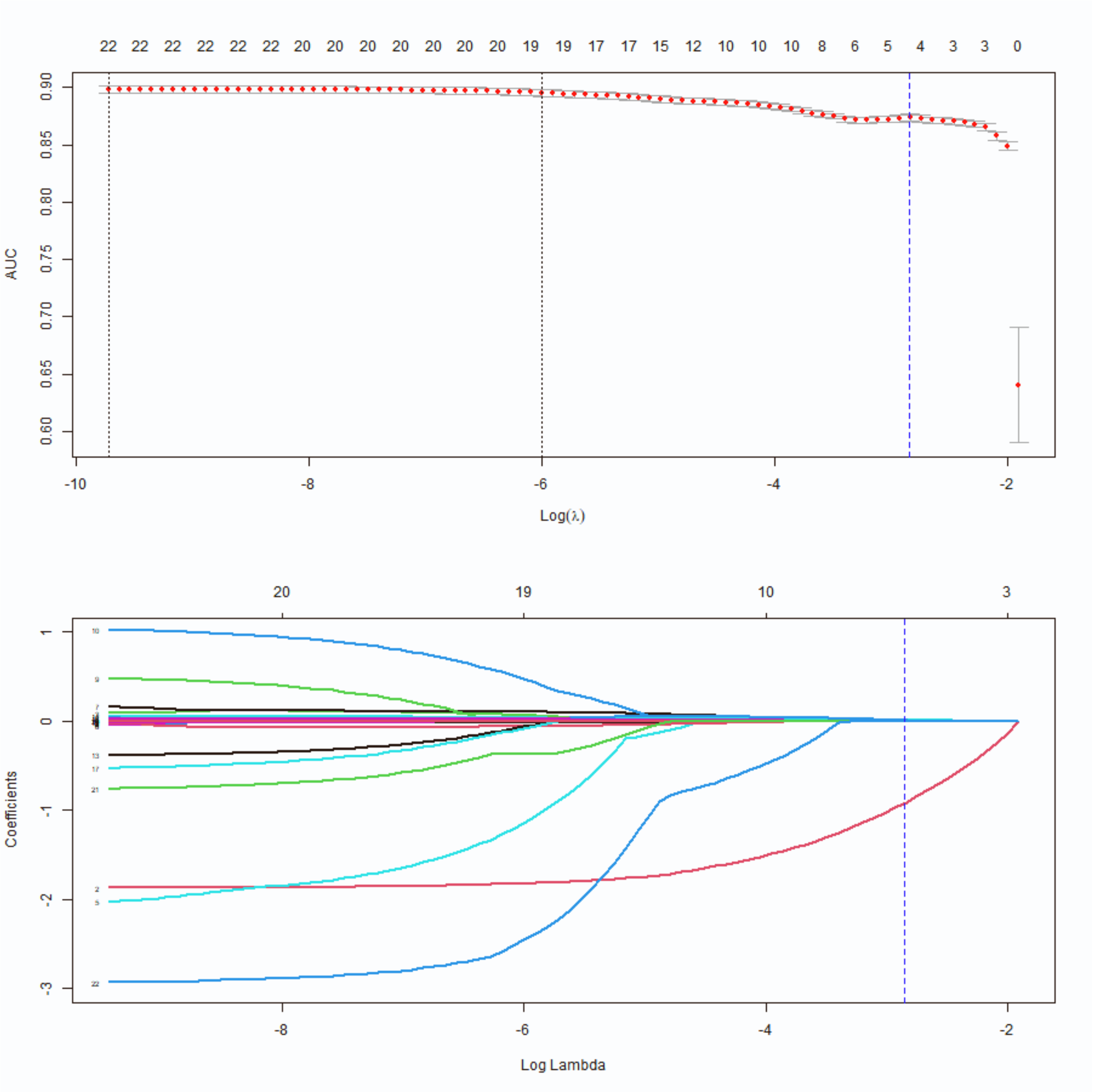

Figure S1. Result of LASSO logistic regression model

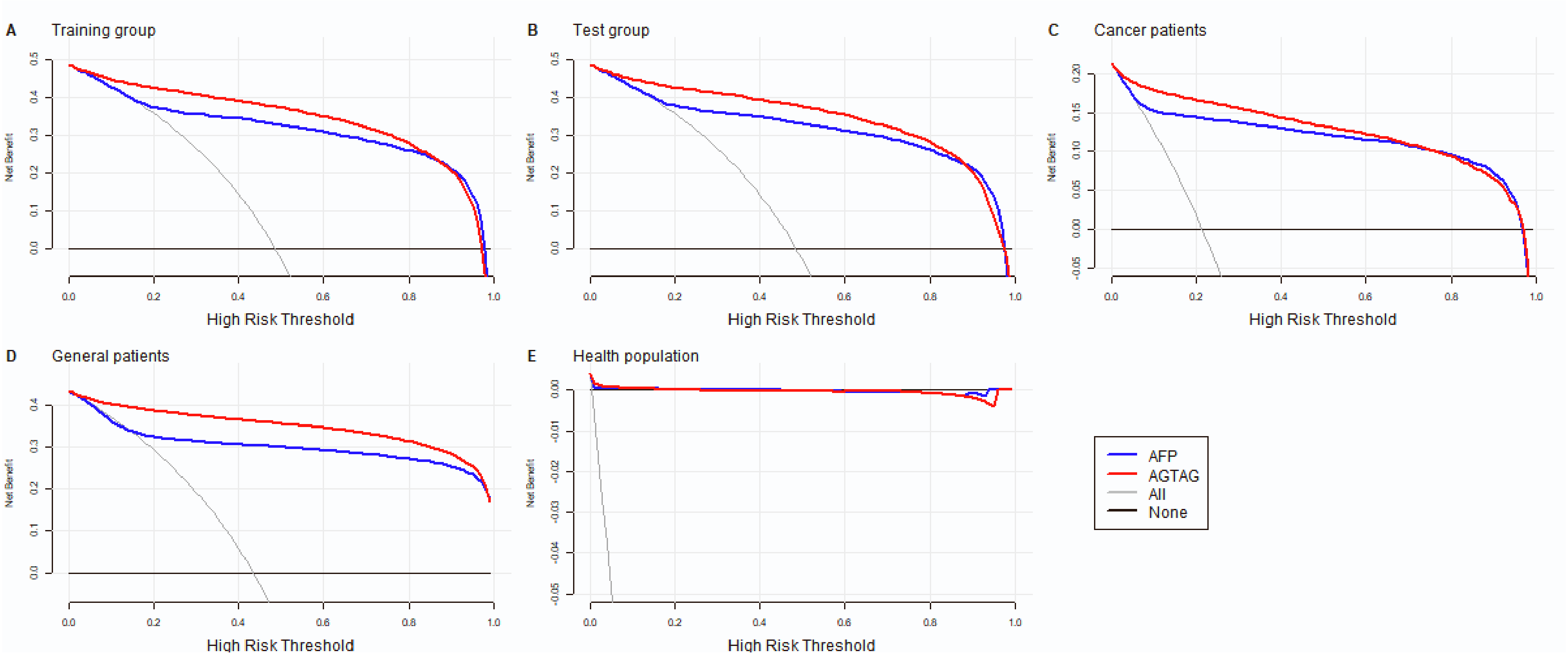

Figure S2. Decision curve analysis of solely AFP versus AGTAG in each cohort

Table S1. variable assignments in LASSO logistic regression model

**OBP Variables**	**Risk Factors**	**Logarithmic Transformation[1]**	**Maximum[2]**	**Minimum[2]**	**Normalization**
**X1**	Age	-			-
**X2**	Gender	-	-	-	male=0, female=1
**X3**	GLO	×	137.50	7.94	√
**X4**	IBIL	√	5.03	0.18	√
**X5**	DBIL	√	6.30	0.00	√
**X6**	ALB/GLO	×	4.70	0.15	√
**X7**	TBIL	√	6.49	0.00	√
**X8**	ApoB	×	5.21	0.00	√
**X9**	AST/ALT	√	3.89	0.17	√
**X10**	HDL-C	×	4.01	0.06	√
**X11**	LDH	√	10.21	3.93	√
**X12**	TBA	√	6.26	0.00	√
**X13**	ALB	×	65.40	15.00	√
**X14**	CRP	√	6.11	0.00	√
**X15**	ALP	√	8.39	1.99	√
**X16**	ApoAI	×	2.96	0.00	√
**X17**	GGT	√	8.07	1.31	√
**X18**	ALT	√	7.41	0.59	√
**X19**	TC	×	23.26	0.10	√
**X20**	AST	√	8.06	1.53	√
**X21**	LDL-C	×	18.32	0.10	√
**X22**	TP	×	163.00	34.57	√
**-**	AFP	√	11.77	0.47	√

Note: [1] logarithmic transformation was applied to positively-skewed measurements and retains the original values of normally-distributed measurements. [2] The maximum and minimum values listed here are calculated from SYSUCC's modeling cohort.

Abbreviations: GLO: globulin, IBIL: indirect bilirubin; DBIL: direct bilirubin; TBIL: total bilirubin; ApoB: apolipoprotein B; HDL-C: high-density lipoprotein cholesterol; LDH: lactic dehydrogenase; TBA: total bile acid; ALB: albumin; CRP: C-reactive protein; ALP: alkaline phosphatase; ApoAI: apolipoprotein AI; GGT: gamma-glutamyl transpeptidase; ALT: alanine aminotransferase; TC: total cholesterol; AST: aspartate transferase; LDL-C: low-density lipoprotein cholesterol; TP: total protein
